# Does cost sharing do more harm or more good? - a systematic literature review

**DOI:** 10.1186/s12889-016-3624-6

**Published:** 2016-09-15

**Authors:** Katarzyna Kolasa, Marta Kowalczyk

**Affiliations:** 1 Health Economics Department, Collegium Medicum Bydgoszcz, Sandomierska 16, 85-630 Bydgoszcz, Poland; 2 Pharmacoeconomics Department, Medical University of Warsaw, Żwirki i Wigury 81, 02-091 Warsaw, Poland

**Keywords:** Kakwani index, Progressivity, Out of pocket payments, Equity

## Abstract

**Background:**

There are positive and negative consequences of the implementation of out of pocket (OOP) payments as a source of the healthcare financing. On the one hand, OOP burden increases awareness of treatment costs and limits unnecessary use of healthcare services. On the other hand, it may prevent the sick from accessing needed care. Consequently there are several aspects that ought to be taken into consideration while defining the optimal structure of OOP payments. The objective of this study was twofold. Firstly, it was to understand what actions are taken to decrease the OOP burden. Secondly, it was to address the question whether the implementation of any form of formal OOP payments may impact negatively upon fairness in financial contribution.

**Methods:**

The literature search was conducted using the Pubmed, Embase, Cochrane Library and Center of Review and Dissemination databases. Only studies which measured the Kakwani index of progressivity in at least two time points were included. Articles written in English published between January 2004 and September 2015 were searched. No geographical restriction was imposed. An increment of more than 0.10 in the Kakwani index was considered as a significant health policy impact.

**Results:**

In total 16 publications were included, of which nine studied attempts to decrease the OOP burden, four described the consequences of the introduction of formal fees, and three covered both topics. Overall, a significant health policy impact was noted in four cases. All of them related to a reduction in the OOP burden, with three and one noting a change towards the progressivity and regressivity of direct healthcare payments respectively. Among jurisdictions which introduced formal fees, none study noted a significant impact on the regressivity of OOP spendings.

**Conclusions:**

In the majority of cases, a health policy impact on the distribution of OOP health payments was insignificant. The reduction of OOP burden cannot be achieved successfully without adequate extension of healthcare coverage or engagement of other sources of healthcare financing. When formal fees are being introduced, protection against catastrophic healthcare payments is needed for the most vulnerable groups.

## Background

According to the World Health Organization there are three main goals for a healthcare system: good health, responsiveness to the expectations of the population, and fairness of financial contribution [1]. While the first objective, overall improvement of health, is self -explanatory the other two require more clarification. Responsiveness addresses the question of how far the healthcare system responds to people’s expectations of it. The concept of fairness can be defined as “the highest possible degree of separation between contribution and utilization”. It demands financial responsibility to vary according to ability to pay, and access to the healthcare system to vary according to healthcare needs irrespective of ability to pay [1].

According to the World Health Organization (WHO), all three of a healthcare system’s ultimate goals should be considered equally valid. Improvement of health alone in society is not sufficient. In order to ensure a health system’s responsiveness and financial protection, the consequences of the actions of health policy, especially with respect to the worst off, have to be thoroughly studied. Even a small absolute risk can be devastating to the health and financial situation of the poorest. Therefore unless the decision makers take into consideration the impact of their health policies on the least disadvantaged groups, all three healthcare system objectives cannot be successfully met. In an era of global crisis and decline in healthcare spending, the financial implications of healthcare policies upon the worst off carry an even stronger meaning [2].

Although the concept of financial protection refers to all sources of healthcare financing, out of pocket (OOP) health payments are of special importance. According to some experts, distribution of the OOP’s burden across society should be considered “a strong test” of the fairness of the healthcare system [3].

When the public share of healthcare financing becomes insufficient, private healthcare expenditures have to compensate for it. If the balance between public and private funds is distorted, the health system objective of financial protection is undermined [4].

The available Organization for Economic Co-operation and Development (OECD) data indicates that OOP spending constitutes roughly 20 % of total health expenditure [5]. The role of direct household healthcare payments goes beyond fiscal consideration. There are positive and negative consequences of cost sharing in the healthcare sector. On the one hand, direct participation of patients in healthcare financing increases awareness of treatment costs and limits unnecessary use of healthcare services. On the other hand, it may prevent the sick and disadvantaged from accessing the care needed [6]. Consequently, OOP health payments ought to be affordable for the worst off. Otherwise differences in access to medical services across society will become apparent and healthcare systems will fail to respond equally to everyone’s needs.

The objective of this paper was to conduct a systematic literature review and investigate how different jurisdictions strive to achieve fairness in financial contribution with respect to OOP health payments. In particular it was to address two questions: What actions do health policy makers take to decrease the burden of OOP spending? Does the implementation of any form of formal user fees impact negatively upon fairness in financial contribution?

To allow comparisons between different time points as well as across jurisdictions, studies which calculated the Kakwani index were selected [7]. The Kakwani Index is the most widely used measure of fairness in financial contributions in the healthcare sector. It addresses the question of how the distribution of OOP health payments departs from proportionality. Equity according to need requires that everyone is entitled to access the healthcare system irrespective of the size of their financial resources. It can only be achieved if healthcare contributions are collected according to the principle of ability to pay. Hence the underlying hypothesis for this study was that for the healthcare system to pursue the objective of fairness in financial contributions it needs to ensure the financial burden is distributed progressively or at least proportionally.

## Methods

The literature search was conducted using the Pubmed, Embase, Cochrane Library and Center of Review and Dissemination databases. The following key words were used: out-of-pocket payments, health expenditures, cost sharing, deductibles, coinsurance and copayments. Each of them was paired with two search words: Kakwani Index and progressivity. In order to address the research questions, studies which measured the Kakwani index of progressivity in at least two time points were included. All articles written in English and published between January 2004 and September 2015 were searched. No geographical restrictions were imposed. Publications limited to the methodological considerations and disease specific studies were excluded.

The systematic review was conducted and reported according to the Preferred Reporting Items for Systematic Reviews and Meta-Analyses (PRISMA) checklist [8]. Selection and review of papers was conducted independently by two reviewers, and disagreements were resolved by consensus.

The following subgroups were constituted for the purpose of the analysis: studies of health policy actions to decrease the burden of OOPs, studies of the consequences of the introduction of a formal system of OOPs, and those which covered both topics. A review of legal changes (health policy impact) during the period studied was conducted to classify each publication into one of the above groups.

The Kakwani index measures the degree to which the distribution of out of pocket payments departs from proportionality. It is calculated as the difference between the Concentration index (C) and the Gini coefficient (G) [7].

$$ {}_k=C\hbox{--} G $$

While the first measures the degree of income-related inequality of a given health variable (in our case OOP payments), the second assesses income inequality. The Kakwani index can take values from minus two to one. Because the concentration index takes a value from minus one to one (when all OOP payments are borne by the poorest and riches person respectively. Whereas the Gini coefficient varies from zero (perfect income equality) to one (perfect income inequality). A positive value of the Kakwani index indicates progressivity. A negative value, on the other hand, means regressivity. If the index equals minus two, the concentration of pre-payment income occurs at the very top of the distribution and the poorest person bears all of the OOP spending. When the Kakwani index equals one, the pre-payment income is distributed equally and all of the OOP payments are borne by the richest person. If, by contrast, direct healthcare expenditures are a proportional source of healthcare financing, the Kakwani index will be zero.

In a graphical way, the Kakwani index can be defined as twice the area between a Lorenz curve and a concentration curve. The Lorenz curve represents income distribution. It plots the cumulative proportion of income against the cumulative proportion of the population ranked according to the income. The more unequal the distribution the further the Lorenz curve lies below the diagonal (the line of perfect equity). The concentration curve represents health variable distribution (such as OOP burden). It plots the cumulative percentage of OOP payments against the cumulative percentage of the population, ranked by income. If OOP expenses are disproportionally distributed amongst poorer (richer) people, the concentration curve will lie above (below) the line of equality. If OOP payments are a progressive (regressive) source of financing, the concentration curve will lie below (above) the Lorenz curve, and the Kakwani index will be positive (negative).

The significance of the change was measured following the approach introduced by Yu et al. (2008). They assumed that an increment in the index of more than 0.10 was considered as significant [9].

Studies of equity in healthcare financing allow for different measurements of ability to pay to be used. Disposable household income or household expenditures are used interchangeably. There are pros and cons for both measurements. Given the potential impact of the choice of ability to pay measurement on results, it was reported separately for each study.

## Results

In total 77 publications which fulfilled the criteria, based on screening of the title, were identified in the databases. As many as 70 were excluded during the abstract review process out of which 37 were repetitions, 17 only reported the Kakwani index for 1 year of observation, five were limited to methodological considerations and seven did not report the Kakwani index at all. Additionally, there were three with limited scope of analysis to a specific group of patients and one was excluded due to non-English language. The remaining seven were selected for the review. A subsequent search of references and grey literature revealed an additional nine studies.

**Fig. 1:**
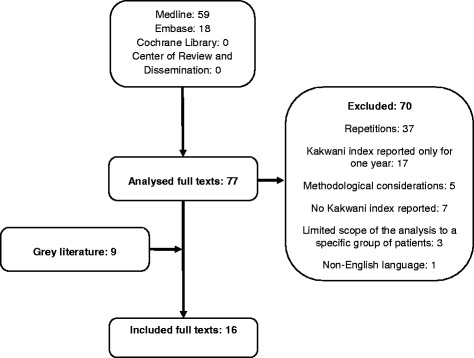
The systematic review flow

In total, 16 publications were included. Figure 1 shows the flow diagram of selection of publications.

Table 1 provides the basic characteristics of all reviewed studies. While eight publications reported income, only seven studies used expenditure as a welfare measurement. In one case, both approaches were tested.

**Table 1:** List of studies included in the systematic review

Author	Jurisdiction	Year of publication	Data source	No of households (year)	Reference
1 Markova N.	Bulgaria	2006	Living Standards Measurement Surveys (LSMS) of the World Bank for 1995 and 2001	2400 (Y 1995–2001)	[23]
2 Hanley G. E.et al.	Canada/British Columbia	2008	BC PharmaNet, prescription drug utilization data for residents registered for BC Medical Services Plan for at least 275 days during each year from 2000 to 2004)	1,700,000 (Y 2000–2004)	[10]
3 Chen M., Zhao Y., Si L.	China	2014	Household Surveys, Heilongjiang province, Northeast China	3841 (Y 2003); 5530 (Y 2008)	[17]
4 Chen M. Chen W. Zhao Y.	China	2012	Household Surveys, Gansu province, China.	3946 (Y 2003); 3958 (Y 2008)	[18]
5 Castano R. et al.	Columbia	2002	Nation-wide cross sectional surveys, the National Department of Statistics (DANE).	26,117 (Y 1984); 28,022 (Y 1994); 9121 (Y 1997).	[14]
6 Krutilova V.	Czech Rep	2013	Household Budget Survey (HBS), Survey on Income and Living Conditions (SILC) and European Health Interview Survey (EHIS)	2765 (Y 2007); 2685 (Y 2008); 2686 (Y 2009)	[22]
7 Võrk A., Saluse J., Habicht J.	Estonia	2009	Estonian Household Budget Surveys	6256 (Y 2000); 6053 (Y 2001); 5721 (Y 2002); 3391 (Y 2003); 3233 (Y 2004); 3601 (Y 2005); 3807 (Y 2006); 3406 (Y 2007)	[20]
8 Dukhan Y et al.	France	2010	French household budget surveys from 1995, 2001 and 2006	10,240 (2006); 10,305 (2001); 9634 (1995)	[25]
9 Baji P. et al.	Hungary	2012	Household Budget Survey, the Central Statistical Office	9058 (Y 2005); 8975 (Y 2006); 8547 (Y 2007); 7650 (Y 2008)	[21]
10 Zare H. et al.	Iran	2014	Iran’s Households Income and Expenditure Survey (HIES), the Statistical Center of Iran (SCI)	342,532 rural; 308,735 urban (Y 1984 - Y 2010)	[19]
11 Smith S.	Ireland	2010	Household Budget Survey (HBS) data for 1987/88,13 1999/2000 and 2004/05.	7705 (Y 1987/88); 7644 (Y 1999/2000); 6884 (Y 2004/05)	[24]
12 Kiss S.	Slovakia	2007	Household Budged Survey, the Statistical Office for 2001–2005 of the Slovak Republic	1600 (Y 2001–2005)	[11]
13 Limwattananon S. et al.	Thailand	2011	Health and Welfare Surveys (HWS) household surveys conducted by the National Statistical Office in 2001, 2003, 2006 and 2007	70,000 individuals (Y 2001 - Y 2007)	[12]
14 Ali SI	Vietnam	2009	Cross-sectional household survey data collected from three provinces: Hai Phong, NinhBinh and Dong Thap	1650 adults; 1101 children (Y 1999)	[13]
15 ÖzlemGöçmez	Turkey	2010	“Household Budget Survey” from Turkish Statistical Institute for years 2003 and 2006	2003- 25,920 households, 2006–8640 households	[15]
16 Yardim M.S. Cilingiroglu N. Yardim N.	Turkey	2014	“Household Budget Survey” from Turkish Statistical Institute for years 2003 and 2006 and 2009	2003-25,920 households, 2006–8640 households, 2009–12,600 households	[16]

Except for the article written by Hanley et al. regarding British Columbia in Canada, all studies utilized household surveys for the purpose of the analysis. In contrast to the others, the Canadian study was based on a prescriptions’ database. The size of study population varied from 1600 households in Slovakia to 1,700,000 households in British Columbia in Canada [10, 11].

Following the approach outlined in the methodology, studies were categorized into three groups. The first one consisted of publications which analyzed health policies’ attempts to decrease the OOP burden. Out of nine studies included in this group, seven focused on the impact of the introduction of universal healthcare coverage, one analyzed the implementation of voluntary healthcare insurance and the last one evaluated the consequences of changes in drug policies (Table 2). The second group consisted of four studies which described the impact of the introduction of OOP health payment regulations on fairness in financial contribution. They covered health policies embracing formal fees for both ambulatory care visits and hospital stays, as well as copayments for drugs (Table 3). Finally the third group gathered the remaining three studies which covered both the implementation of public and private healthcare insurance as well as the introduction of formal fees for healthcare visits (Table 4).

**Table 2:** The description of health policies aiming at OOP burden reduction

Jurisdiction	Health policy objective in the studied period	Cost sharing mechanism		Reference
		Outpatient/Inpatient services	Pharmaceuticals	
1 Canada	Decrease the OOP burden regarding pharmaceutical spending for least disadvantaged	NA	Shift from age-based to income-based eligibility drug reimbursement system: 1. before 2002; 100 % drug coverage for social assistance recipients, 100 % coverage with pharmacists’ dispensing fees for seniors and fixed-deductible coverage for ‘catastrophic’ drug expenses for others 2. In 2002; prescription fees for seniors with cap on spending, others remained unchanged 3. from 2003; three age-income groups, co-insurance varies from 0 to 30 %, family deductibles- from 0 to 3 % of household gross income, max OOPs- from 0 to 4 % of household gross income	[10]
2 China	Decrease the OOP burden after the introduction of insurance based healthcare system.	There are two types of healthcare insurance for city dwellers. Urban Resident’s Basic Medical Insurance (UWBMI) for employees and Urban Resident’s Basic Medical Insurance (URBMI) for the unemployed, children, students, and elderly persons without pensions were introduced. In the UWBMI, employees and employers contribute 2 % and 6–8 % of salaries respectively. The URBMI is funded by individuals with appropriate subsidies granted by government. In 2003 New Rural Cooperative Medical Scheme (NCMS) for rural workers were established (92 % coverage in 2007).	NA	[17, 18]
3 Columbia	Decrease the OOP burden after the introduction of insurance based healthcare system.	In 1993 National Social Health Insurance System (NSHIS) was established: 1. Employed and self-employed were financed solidarly by employees and employers (in total 12 % of salary). It covered all first-degree family members of those who contribute and pensioners. 2. Poor were financed by taxes and solidarity contribution from other insurance funds. The poor was defined by set of criteria such as labor market participation, income, educational attainment, family structure, access to water and sanitation and others. Interventions are grouped by categories of medical care and levels of complexity.	NA	[14, 32]
4 Iran	Decrease the OOP burden after the introduction of insurance based healthcare system.	Healthcare reform steps: 1. development of primary health care (PHC) networks and medical facilities (1990–94), 2. the introduction of health insurance (1994–99), 3. Further development and improvement of healthcare coverage (2000–04), 4. decreasing inequalities in health expenditures (2005–09)	NA	[19]
5 Thailand	The extension of universal healthcare coverage	Since October 2001, Universal health insurance system: the curative package (ambulatory and hospitalization service), the high-cost care package, and the promotive and preventive package. The B 30 copayment was introduced in 2001 (equivalent to US$ 1 in 2010) per ambulatory visit or hospital admission. It was abolished in 2006. The total number of insured rose from 33 % in 1991 to 71 % in 2001 and 98 % in 2007. In 2007, the universal coverage was the biggest insurer (75 % of total population), Social Security Scheme for private employees (13 %), Civil servants for public employees (8 %) private health insurance (2 %).	In 2003 a universal access to antiretroviral drugs was established.	[12]
6 Turkey	Extension of free of charge healthcare for low income inhabitants (green card holders)	In 1992 a Green card system was established for income below one-third of the base wage rate (ca 18 % of population in 2007). It allowed a free access to inpatient care. In 2004 it was extended to cover alllevels of healthcare except for 20 % co-payment for pharmaceuticals. One year later, Green Card holders were given access to outpatient care and pharmaceuticals. In 2008, they have formally joined Universal Health Insurance. By 2011, about 85 % of the poorest decile was covered by the Green Card or another insurance scheme. Non-Green card holders pay 8 TL (€3.6) and 15 TL (€6.8) for outpatient services in public and private hospitals, respectively unless they have referral from a GP. Primary care services are free of charge. After 2003, additional copays may apply if the cost of care in a private hospital is higher than public reimbursement. Informal payments are estimated at 5.2 % of total OOP expenditure.	20 % of prescription charges for all active workers including Green Card holders; retirees pay 10 %. Since 2004, 333 jumbo referencing groups established. A reimbursement for any product set at the level of the cheapest in the group plus 15 %. Patient pays the difference between reimbursement and the actual price of the drug.	[15, 16, 38, 39]
7 Vietnam	The role of Voluntary Health Insurance in broadening the access to healthcare system	Since 1991, healthcare services were covered mainly through OOPs. After healthcare reform in 1992, three groups of beneficiers were established: 1.eligible for Compulsory Health Insurance (public sector, workers of private companies companies with over ten employees) 2. eligible for Voluntary Health Insurance (employed not included in 1, self-employed, dependend of those in group 1, school children and other students) 3. Not eligible for Compulsory Health Insurance and too poor for VHI. Out of 76 mln of Vietnamese in the group 2, 3.6 mln had VHI and 33.4 mln still paid fully OOPs. Since 1998, insured patients are obliged to make a copayment of 20 % of the total costs of care provided. An annual ceiling of half the minimum annual salary was introduced as well.	NA	[13]

**Table 3:** The description of consequences of OOP healthcare payments’ introduction

Jurisdiction	Health policy change in studied period	Cost sharing mechanism		References
		Outpatient/Inpatient services	Pharmaceuticals	
1 Czech Rep	Introduction of formal fees	Until the end of 2007, outpatient and inpatient services were free of charge. Since 2008, formal OOPs exists; a flat fee of 30 CZK (€1.2) per doctor visit, 60 CZK (€2.4) per hospital day as well as spa hotels, 90 CZK (€3.6) per ambulatory visit outside of working hours. An annual ceiling of CZK 5000 (€200) for expenses related to doctor visits and drug costs was introduced. Since 2009, a new ceiling of CZK 2500 (€100) for those below 18 and above 65 was launched. A flat fee per doctor visit for children was eliminated. Since 2012, a flat fee for hospital and spa stay was reduced to 100 CZK per stay (€3.92 EUR). The dental care is paid by OOPs too. Some groups such as poor, pregnant woman, chronically ill children, patients with infectious diseases are exempted from formal OOPs. (Exchange rate used; 1 EUR = 250 CZK)	Until 2008, some form of co-payments existed. A prescription fee of 30 CZK (1.18 EUR) per item was introduced in 2008. Since 2009, a difference between actual and reimbursement price is paid out of pocket if it is higher than prescription fee.	[3, 22]
2 Estonia	Introduction of formal fees	Since 1995, a fee of €0.30 (EEK 5) per first initial outpatient consultation at public hospitals and/or health centers exists, a free price setting for specialists. Since 2002, no fees for GP visits (except for home GP visit which is €3.2) but ambulatory specialist care at maximum fee of €3.20 (EEK 50) unless a referral within the same institution or specialty is granted. Hospital fees are implemented at 1.6 EUR per day, for up to 10 days per episode of illness. Some exemptions for children, pregnant woman and emergency care apply. (Exchange rate used; 1 EUR = 15.6 EEK)	Co-payment consist of a prescription fee of €1.30 plus the difference between actual price and reimbursement level. The general reimbursement rate is 50 % of the pharmaceutical price up to a maximum reimbursement of €12.00 (EEK 200) per prescription. The reimbursement of drugs for chronic disease, children, seniors and disabled is higher, up to 100 % .	[40, 41]
3 Hungary	Introduction of formal fees	To limited extend, some form of copayments already existed since 1989 (medical devices, spas, specialist treatment outside of standard patient’s pathway etc.). Since 2007 formal co-payments were introduced; app €1 per ambulatory visit and per hospital day. After the referendum held in 2008, they were abolished.	Since 2007 reimbursement rates have been decreased from 50 to 25 %; from 70 to 55 %; and from 90 to 85 %. For drugs with a special reimbursement of 90 %, three levels of coverage was established: 50, 70 and 90 %. For drugs fully reimbursed, a minimum €1 (300 HUF) fee per prescription was introduced. For special attentionpatients eligible for free of charge drugs, a monthly limit of 40 EUR was established. OOPs apply above that sum. Eligibility for special attention is defined by GP. (Exchange rate used; 1 EUR = 250 HUF)	[21, 34]
4 Slovakia	Introduction of formal fees	The formal copayments were introduced in 2003. Since then, app. €0.66 is paid per doctor visit and app. 1.66 EUR per hospital day, app €1.99 per emergency care visit,€0.07 per km for ambulance transport and between €4.98 and 7.30 per food and accommodation in spas. In 2006, user fees for a doctor’s visit and daily hospital stay were abolished. (Exchange rate used; 1 EUR = 40.03 SKK)	Until 2003, some form of co-payments existed. Since then €0.5 EUR prescription fee has to be paid. It was reduced to €0.17in 2006. If there is a difference between the price of the drug and the reimbursement level, patient has to cover it as well.	[11, 42]

**Table 4:** The description of health policies aiming both OOP healthcare payments’ introduction and at OOP burden reduction

Jurisdiction	Health policy change in studied period	Cost sharing mechanism		References
		Outpatient/Inpatient services	Pharmaceuticals	
1 Bulgaria	1. Introduction of universal healthcare insurance system 2. Implementation of formal fees	The healthcare insurance act of 1998 converted the Bulgarian health system into a health insurance system. Since 2000 formal co-payments at 1 % of the minimum wage for GP and outpatient visit, 2 % of the minimum wage for the first 10 days of the hospital stay (no fee for a subsequent hospitalization during the year), emergency care without co-pays, full price for specialist care and other services outside the standard patient pathway. User fees apply to all patients with some exceptions: children, pregnant women, unemployed individuals, those with income below a certain threshold, chronically sick patients and some other groups.	There is a Positive Drug List which shortlist full coverage for outpatient, inpatient settings as well as treatment for oncological, rare, infectious diseases as well as AIDS. Drugs outside Positive List have to be fully paid out of pocket.	[29]
2 France	1. Introduction of public complementary health insurance coverage for certain groups introduced 2. Implementation of formal fees	In general, healthcare insurance coverage varies from 100 % for hospital care to 70 % for ambulatory care and 60 % for medical auxiliaries as well as for laboratory tests. Full coverage exists for long-term illnesses, pregnant woman (after 5th month) and others. A system of copayments; since 2004, an extra co-payment for direct access to specialists or other GPs without remission (40 % of the standard SHI tariff). a flat-rate catering fee of €18 per day for accommodation in hospital. Since 2005, €1 on every physician visit, biological test and radiograph up to a ceiling of €4 per day and €50 per year has been introduced. Since 2006, patients have had to pay a flat rate of €18 for care with a statutory tariff over €91. VHI covers cost sharing without flat fees (other exemptions apply as well). The VHI population’s coverage increased from 50 % in 1970 to 88 % in 2006. A free public complementary health insurance (CMU) and a voucher scheme (ACS) for those who cannot afford VHI were established in 2000 and 2004 respectively.	Reimbursement rates varies from 15, 35, 65 or 100 %. On the average rate of reimbursement for drugs is estimated to be 73 %. There is a fee of €0.5 is charged for every drug package up to a ceiling of €50 per year .	[30]
3 Ireland	1. The expansion of GP Visit Card accessibility 2. A decline of 95,000 in the number of Medical Card holders (1997–2005)	For medical card holders (eligibility is set based on income and age) there is a free of charge GP, hospital and dental care, drugs, medical appliances and others. Non-medical card holders pay out of pocket for GP visits (from €50 to €90), consultants’ fees, €66 for hospital stay per day up to €660 per year. Based on a referral for inpatient and outpatient services, no charges are levied for diagnostic tests. Private health insurance covers fully OOPs for inpatient care and outpatient services to some extent. The costs of dental and optical care is reduced for Treatment Benefit Scheme holders (operated by the Department of Social and Family Affairs for those who pays Pay-related social insurance). The number of medical cardholders decreased from 37 % in 80-ties to 30 % in 2007. Since 2005, for those with income up to 50 % (change from 25 %) higher than the ceiling for a Medical Card, a free of charge GP visits’ system (GP Visit Card) was introduced. The evolution of private health insurance from 4 %- 1960 to 35 % -1987	For medical card holders - free of charge, for others - up to €90 per month. For chronic long-Term Illness Scheme, open to individuals with one of a number of predefined chronic conditions - covers the costs of all necessary pharmaceuticals, medicines and appliances Others bears full cost of the drug but they should apply for a Drugs Payment Scheme (DPS) which limits out-of-pocket expenditure for an individual or family to no more than certain ceiling (for example in 2014; €144) per calendar month for prescribed pharmaceuticals, medicines and appliances. DPS replaced Drugs Refund Scheme in July 1999. DRS operated on similar principles as DPS.	[24, 35, 36]

Out of the nine publications included in the first group, three reported significant improvement in progressively of OOP payment distribution measured by the Kakwani index during the study period (Table 5). This was the case for British Columbia in Canada, Thailand and Vietnam [10, 12, 13]. The first one differed from the others by the fact that OOP spending was the most regressive source of healthcare financing at baseline i.e. before the reform took place. Although only limited to the drug policy, it was the largest and most significant reduction of regressivity across all of the studies reviewed. In Thailand, the introduction of a universal health insurance system lead to significant improvement of the Kakwani index [12]. Conditional on the methodological approach taken, the Vietnamese study indicated that voluntary healthcare insurance (VHI) significantly improved the progressivity of OOP payments compared to non-VHI healthcare users as well [13].

**Table 5:** Kakwani index of progressivity in selected publications

		Group		Kakwani index			Ability to pay		Reference
			Year	OOP	Private health insurance	Total healthcare financing	Household expenditures	Disposable income	
1	Canada	1	2001 (non seniors)	−0.373	−0.100			x	[10]
			2001 (seniors)	−0.299	NA				
			2004 (non seniors)	−0.253					
			2004 (seniors)	−0.078					
			2004	−0.195	−0.099	−0.087			
2,3	China Heilongjiang province		2002 (urban)	0.088	NA			x	
			2002 (rural)	0.075					
			2007 (urban)	−0.020					
			2007 (rural)	0.027					
	China Gansu province		2002 (urban)	0.0455	0.0854	0.0431	x		[18]
			2002 (rural)	0.0448	0.0810	0.0148			
			2007 (urban)	0.0488	0.0089	0.0351			
			2007 (rural)	0.0086	0.2534	−0.0226			
4	Columbia		1984	−0.009	NA		x	x	[14]
			1997	0.003					
5	Iran		1984 (urban)	0.455	NA		x		[19]
			1984 (rural)	0.443					
			2010 (urban)	0.446					
			2010 (rural)	0.417					
6	Thailand		2000	−0.150	−0.362	−0.004		x	[12]
			2002	−0.076	−0.391	0.001			
			2004	−0.076	−0.323	0.034			
			2006	−0.045		0.041			
7,8	Turkey		2003	−0.147	NA			x	[15]
			2006	−0.152					
			2003	0.079			x		[16]
			2006	0.009					
			2009	−0.028					
9	Vietnam		Insured (1999)	from (−0.244) to (−0.065)	NA		x		[13]
			Uninsured (1999)	from (−0.242) to (−0.173)					
10	Czech Republic	2	2007	−0.084	NA		x		[22]
			2008	−0.125					
			2009	−0.114					
11	Estonia		2000	−0.300	NA	0.032	x		[20]
			2007	−0.379		0.005			
12	Hungary		2005	−0.220	NA			x	[21]
			2006	−0.224					
			2007	−0.220					
			2008	−0.215					
13	Slovakia		2001	−0.170	NA	0.020		x	[11]
			2005	−0.210		−0.010			
14	Bulgaria	3	1995	−0.320	NA	−0.258	x		[23]
			2001	−0.396		−0.316			
15	France		2001	0.043	−0.248	NA	x		[25]
			2004	0.046	−0.254				
16	Ireland		1987/1988	−0.010	0.080	NA		x	[24]
			1999/2000	−0.100	0.060				
			2004/2005	−0.108	−0.032				

Apart from one, the remaining publications in the first group did not report any significant decrease of OOP burden. Among them were studies regarding Columbia, Turkey, China and Iran [14–19]. While the Kakwani index improved slightly for Columbia, the opposite was found for Turkey. Despite minor differences in the methodological approach towards the calculation of the income variable both studies provided similar trends for changes in the Kakwani index in Turkey. Not only did the trends for both China and Iran indicate change towards less progressive OOP payment distribution but they lay outside the range for others. Apart from a significant change in the regressivity of the Kakwani index for the Chinese urban cohort, all other results were found insignificant.

As far as the second group is concerned, none of the studies reached significance in the change to the Kakwani index (Table 2). Among the publications included, Central Eastern European settings prevailed [20–22]. The implementation of formal user fees made OOP health payments most burdensome in Estonia [20]. A similar health policy change cannot be neglected with respect to the Slovak households either [11]. Comparison of the Kakwani index revealed that Hungarian OOP payments were the second most regressive source of healthcare financing across the jurisdictions studied [21]. In the Czech Republic the introduction of formal user fees affected the poorest to the greatest extent in the first year, the situation however improved in the subsequent year. It must be mentioned however that Czech co-payments for General Practitioners (GPs) were comparable to those in Estonia. In the majority of cases across the jurisdictions studied, specialists’ fees varied depending upon referral from GPs. Among the protection mechanisms for the most vulnerable, the exemption mechanism prevailed. A ceiling on copayments was introduced in the Czech Republic and Estonia as well as Hungary [20–22].

In similar fashion to the second group, none of the studies in the last group produced significant results regarding changes in the Kakwani index in the period studied. Among the publications included in the third group, there were studies of Bulgarian, Irish and French reforms [23–25]. The results were mixed. In the first case, the introduction of healthcare insurance alongside formal fees for healthcare visits worsened the OOP burden. The trend towards regressivity of OOP spending continued throughout the observation period in Ireland as well [24]. It has to be underlined that the Irish data allowed for the longest follow up. In France, by contrast, the Kakwani index stayed almost unchanged [25]. Although OOP payments remained a positive source of healthcare financing throughout the observation period there, the Kakwani index for VHI remained negative which balanced the progressivity of OOP payments to some extent.

As far as the choice of proxy for the ability to pay is concerned, both household disposable income and household expenditure were utilized so that half of the studies used the first and the other half the second measurement. No clear pattern of impact related to choice on the results could be distinguished.

## Discussion

Among the 16 studies included, a significant health policy impact was only achieved in four cases, and all of these related to the reduction of the OOP burden. Among them were three studies which revealed a change towards progressivity. The remaining one was reported for China and surprisingly indicated a trend towards regressivity of OOP payments. As the Kakwani index for the Chinese study lay outside the range of the others, its results have to be treated with caution.

None of the studies related to the introduction of formal fees for healthcare visits noted a significant impact on OOP payment distribution.

The majority of health policy attempts on the reduction of OOP burden were studied in developing countries. Interestingly enough, publications regarding the consequences of the introduction of formal fees for healthcare visits concerned healthcare reforms conducted in Central Eastern European (CEE) settings.

As far as the first study objective is concerned, the systematic literature review that was performed provided two key valuable insights into the effects of health policies against excessive OOP burden.

First of all, revealed evidence indicated that the reduction of a household financial healthcare burden could not be achieved without adequate involvement of indirect sources of healthcare financing. This was proved with the examples from both Columbia and Bulgaria [14, 23]. In the Columbian case, the health care reform was based on social insurance. Due to a slow uptake in the implementation of the new scheme, the achievement of equal distribution of OOP payments was very limited [14]. Seven years after the reform’s implementation, as many as 40 % of the eligible poor still did not have healthcare insurance coverage [14]. Additionally, the scope of reimbursement remained very limited to primary care services. In similarity with the Columbian example, a slow uptake of healthcare insurance coverage was noted in Bulgaria [23]. Public sources still constituted only 57 % of total healthcare financing 7 years after the reform took place [26]. Furthermore as many as 25 % of citizens remained uninsured in 2011 and 19 % in 2013 [27, 28]. It is estimated that up to half of private expenses could be caused by underfunding of the healthcare system from public sources in Bulgaria [29].

Secondly, the reviewed evidence provides some indication that the negative consequences of the households’ direct engagement in the healthcare financing could be mitigated with voluntary health insurances (VHI). A notable example in this regard is France. The Kakwani index of OOP payments was positive in both years of the study. The potential explanation for such a result could be the popularization of VHI, the key role of which is to provide reimbursement of user fees. VHI accounted for 13 % of total expenditure on health and covered more than 90 % of the population in 2007 [30]. There are, however, risks involved with reliance on VHI as a key contributor to the healthcare budget. On the one hand, VHI does ensure progressivity of OOP payments. On the other, it is a regressive source of healthcare financing on its own. According to the available studies, socio-demographic differences favoring the well-off among the consumers of VHI prevail in France. In addition to the steady growth of the insurance premium, the scope of VHI’s coverage has only broadened to a limited extent. According to the available data, VHI turnover increased by 48 % while benefits only broadened by 32 % in the same period of time [30]. Having acknowledged the access issue, the French government launched numerous solutions for poor groups of society, including public complementary health insurance (CMU) and a voucher scheme (ACS) for those who cannot afford VHI (Table 4). It remains to be seen whether these solutions are effective enough to improve equity in access to the healthcare sector.

The Irish example could provide additional insight into how to overcome economic barriers for poor households when discussing the importance of VHI for the reduction of the OOP burden [31]. According to legal regulations, Irish insurers must not vary coverage or premium by age or gender, nor current or prospective state of health or any other risk factor. There are additional principles such as open enrollment, lifetime cover and regulated premiums which minimize access barriers for the least disadvantaged. Obviously, it is not possible to conclude whether these particular features of the Irish system made the Kakwani index of VHI progressive or almost proportional in the period studied. The so called community rating system certainly has pros and cons. On the one hand, it ensures that unhealthy poor individuals are not faced with catastrophic OOPs. On the other, it does not prevent the healthy poor from subsidizing the health costs of others. The Vietnamese example indicates that the overall balance could be positive [32]. If the financial contribution is simply lower than the potential costs of healthcare otherwise purchased on the private market, VHI can reduce the financial burden on poor households. The Vietnamese results revealed that the distribution of OOP payments was more equitable among those who purchased VHI than the rest of the population.

As far as the second research objective is concerned, the conducted literature review addressed the question as to whether the implementation of any form of OOPs may impact negatively upon fairness in financial contribution. Oddly enough, it revealed that the introduction of formal user fees for healthcare visits did not affect the distribution of OOP health expenses to a significant extent. Nevertheless, the majority of jurisdictions subsequently abolished them anyway. It is however not surprising, if one takes into account the fact that four out of five jurisdictions did introduce formal user fees without any protective mechanism against excessive direct healthcare expenditures. In Hungary, the system for privileged groups became even more restrictive prior to the implementation of formal fees. The right to free of charge access to drugs was replaced by a monthly personal budget of up to HUF 12,000 (€45) to cover private expenses associated with the treatment of chronic diseases, along with HUF 6000 (€24) for acute problems [33]. Expenses above these limits were to be covered out of pocket.

The Czech Republic was the only jurisdiction which actually introduced a ceiling for formal user fees’. Even there however, there were some challenges with implementation. Firstly a cap of 200 EUR was established for expenses related to doctor visits and drug costs. It turned out however that only 0.2 % of those insured exceeded this limit in 2008. To adjust further for the real burden of OOP payments, the cap was limited to 100 EUR for the most vulnerable groups of patients in 2009 [34]. Although it is unclear how the introduction of the ceiling translated into a reduction in the burden of OOPs, it has to be admitted that the Kakwani index measured after the introduction of formal fees was the lowest in the Czech Republic across all five jurisdictions included in the systematic review which did introduce formal user fees. Ceilings on spending on OOPs exist in both France and Ireland as well. Interestingly enough, in each of these jurisdictions the Kakwani index was higher compared to any of the five countries which launched formal fees during the observation period.

The importance of exemption from direct healthcare payments for the least disadvantaged can be illustrated by the Irish example. In principle, the poorest were entitled to free of charge healthcare. The so called system of medical cards covered roughly 30 % of the population [35, 36]. From 2001 until 2009, eligibility was broadened to those above 70 years of age irrespective of income as well. Before this happened, due to legal changes a decline in the number of medical card holders (roughly 94,000) was observed. According to some experts, this decrease was actually responsible for the Kakwani index being in favor of the better off in 2004/2005 [24].

Although productive in the number of conclusions drawn, the systematic review conducted was not free from limitations. There are at least four reasons which warrant some caution in the ability to generalize the findings across jurisdictions. Firstly, the scope of the study did not include any other measures of OOP distribution such as fairness in financial contribution (FFC) or catastrophic health care expenditures. Secondly, it excluded publications which presented Kakwani index less often than at two separate time points. Thirdly, only English written papers published in the last 10 years were reviewed. Fourthly, due to the variety of the local settings across included studies, some caution should be executed while interpreting the results of the conducted systematic review. The political, social and economic circumstances under which a given health policy was implemented, inevitably had some impact on its outcome. Hence the generalizability of our study findings is limited to some extent.

Finally, there are various forms of direct patients’ participation in healthcare financing such as coinsurance, copayments or deductibles. Although they differ from each other in terms of the division of the financial responsibilities between patient and healthcare service provider, the scope of this particular systematic review was limited to the study of the overall distribution of OOP payments across income groups only. Hence, potential differences in the impact of various forms of household engagement in the healthcare financing on the equity in the healthcare financing could not be distinguished.

Regardless of the above mentioned limitations, the systematic review that was carried out provides a couple of key recommendations for both researchers and health policy makers interested in the analysis of the OOP burden in the healthcare sector.

Turning firstly to future researchers analyzing equity of healthcare financing, the conducted systematic literature review underlines how crucial it is to understand the context in which the Kakwani index is measured. This can be illustrated with examples from China and Iran [17–19]. Irrespective of reforms, direct household spending still remained a key source of healthcare financing there. The observed progressive distribution of OOP payments is driven mainly by the healthcare consumption of the richest, who purchase healthcare services on the private market. Poor groups cannot afford it and do not seek medical help. Hence the positive value of the Kakwani index should be interpreted as an issue with access to the healthcare system rather than equitable distribution favoring the least disadvantaged.

The Canadian study is another interesting example of the importance of the context of the analysis of progressivity of OOP payments [10]. In fact, the improvement of the Kakwani index arose from the decline in public subsidy for high-income seniors rather than an increased benefit for low-income non-seniors. Namely, as a consequence of a change from age-based to income-based subsidies, there was a reduction of public coverage for high-income seniors. It was estimated that the public subsidy for senior households with incomes above the 95th percentile was reduced from approximately 65 % to less than 20 % of their drug expenses [10]. The change towards a more progressive distribution of OOP spending was not accompanied, however, by any redistribution effects to the benefit of low-income households. Hence the study of the Kakwani index of progressivity alone does not provide a full picture. In order to understand the consequences of each policy change, studies on the utilization patterns of healthcare services have to be undertaken as well.

Turning secondly to health policy makers evaluating alternative attempts to reduce the OOP burden, the conducted systematic literature review indicates that to achieve progressivity in health financing the share of regressive sources, particularly out-of-pocket payments, can be reduced or replaced by adequate funds from indirect financial sources. The study from Thailand provides a very inspiring example in this regard. The introduction of a tax-funded health insurance scheme provided coverage for 75 % of the population who were previously not entitled to any other Benefit Schemes. The increase in financing from direct taxes (from 18 % in 2000 to 24.5 % in 2006) and social health insurance (SHI) contribution (from 5.3 % in 2000 to 8.9 % in 2004) lead to the reduction of the OOP burden (from 33.7 % in 2000 to 23.2 % in 2006) and an improvement of the Kakwani index from −0.150 to −0.045 [12].

Taking the health policy makers’ perspective into account, the performed systematic review indicates clearly that a universal system based on health tax or SHI contribution needs to be implementable first to effectively improve fairness in financial contribution.

The example from Bulgaria illustrates that too slow an uptake of SHI did not manage to diminish the OOP burden throughout the observation period. Instead of being a tool to navigate the demand towards more appropriate channels, direct healthcare expenditures still remained a very important source of healthcare financing. In 2009 formal payments alone accounted for 35.3 % of total health expenditure in Bulgaria [37].

As an alternative solution to the increase of healthcare financing from indirect sources, health policy makers can turn to the introduction of private healthcare insurance as well. Indeed the evidence available provides some support for such a solution. However, it should be underlined that VHI acts against the key principle of user fees, which is to increase awareness regarding the real cost of specific treatments. Cost-sharing mechanisms aim to ensure optimal allocation of available healthcare resources by directing demand towards the most effective healthcare services designed for specific health problems as well. Thus, the elevation of patients’ fees might be counterproductive towards allocative efficiency of the healthcare system. Some jurisdictions have initiated actions to mitigate such risks. The French authorities introduced an additional fee for patients who choose to divert their treatment path from standard procedures. Moreover, some financial incentives have been introduced for insurance companies not to cover certain patients’ fees within VHI [30]. In similarity with the French example, the Irish authorities introduced specific regulations regarding voluntary healthcare insurance [36]. While hospital fees are mainly covered by private health insurance, GP fees are only reimbursed by VHI to a certain extent.

If the health policy objective is to improve fairness in financial contribution by the elimination of informal fees, the review of available literature indicates that there is little chance of improving progressivity after the introduction of formal cost sharing. The Hungarian example could provide interesting learning in this regard. After the implementation of formal fees, the regressivity pattern of OOP payments may still remain a challenge in Hungary. Some reduction of inequity in the distribution of informal gratuities indeed took place (the Kakwani index changed from −0.20 to −0.12), however the formal fees turned out to be a regressive source of healthcare financing (the Kakwani index decreased from −0.004 to −0.096) [20]. Hence to avoid further regressivity of OOP payments with cost sharing schemes, protection against the excessive burden of OOPs for the least disadvantaged groups of society have to be thoroughly considered.

## Conclusions

Although limited to only 16 examples, the literature review that was carried out provides an interesting insight into real life examples regarding the impact of different health policies on the distribution of direct healthcare expenditures. Achieving fairness in financial contribution with respect to OOP health payments is not an easy challenge. A healthcare financing system is built on several interconnected pillars, and a healthcare reform focused on one of them will never be successful unless the impact on others is taken into consideration. There is still a lot of room for improvement among both the most and least developed countries. No “one size fits all” solution has been discovered. Nevertheless, learning from the successes and failures of others must be regarded as an important lesson prior to any healthcare reform implementation.

## Abbreviations

C: Concentration index
G: Gini coefficient
GP: General practitioner
OOPs: Out of pocket payments
PRISMA: Preferred Reporting Items for Systematic Reviews and Meta-Analyses
SHI: Social health insurance
VHI: Voluntary health insurance
WHO: World Health Organization
